# Rapid Non-Destructive Assessment of Aquatic Products Freshness by Gas Sensor Based on Morphology-Controlled SnO_2_ Hollow Nanosphere

**DOI:** 10.3390/foods15122123

**Published:** 2026-06-12

**Authors:** Han Liu, Yingkun Dong, Haixia Zhou, Weihao Wu, Ziliang Fan, Cheng Zhao, Yongheng Zhu

**Affiliations:** 1 College of Food Science and Technology, Shanghai Ocean University, Shanghai 201306, China; m230351145@st.shou.edu.cn (H.L.); 19818505418@163.com (Y.D.); d250400135@st.shou.edu.cn (H.Z.); d240400095@st.shou.edu.cn (W.W.); 18888820272@163.com (Z.F.); 2 Henan Railway Food Safety Management Engineering Technology Research Center, Zhengzhou Railway Vocational & Technical College, Zhengzhou 450000, China

**Keywords:** SnO_2_ hollow nanosphere, gas sensor, trimethylamine detection, rapid non-destructive assessment, aquatic product freshness

## Abstract

Trimethylamine (TMA), a characteristic volatile biogenic amine generated during aquatic product spoilage, has a concentration that quantitatively reflects product freshness. Therefore, developing a rapid and accurate method for TMA detection is important for food safety control. Herein, this study synthesized high-performance hollow SnO_2_ nanospheres via a hydrothermal method, aiming to develop a rapid, non-destructive gas sensor for TMA detection and evaluate its feasibility for assessing aquatic product freshness. The material exhibited a high response (Ra/Rg = 10.5@100 ppm), rapid response-recovery kinetics (10 s/20 s), and good selectivity. These properties were attributed to the high specific surface area, efficient gas diffusion channels, and abundant active sites provided by the hollow structure, which enhances the sensor’s response rate. Ultraviolet–visible diffuse reflectance spectroscopy further showed that the hollow structure narrows the bandgap of SnO_2_, which may facilitate electron transfer and contribute to the enhanced response to TMA. In practical applications, a MEMS sensor based on SnO_2_ hollow nanospheres successfully detected TMA concentration changes from sea bass during 0–8 days of refrigerated storage, demonstrating its potential reliability for rapid freshness assessment of aquatic products and providing a technological route for quality evaluation.

## 1. Introduction

Aquatic products are popular among consumers for being high in protein but low in fat. However, they are easily spoiled by microorganisms during storage and transportation [1]. Trimethylamine (TMA) is a typical volatile organic compound produced from spoiled aquatic products. Its level directly reflects freshness: below 1 mg/100 g for fresh samples, 1–5 mg/100 g for early spoilage, and above 6 mg/100 g for severely spoiled ones [2]. At room temperature, TMA is a colorless and volatile gas with an unpleasant fishy smell. As its concentration rises, the odor becomes pungent, similar to high-concentration ammonia [3]. TMA readily enters the human body through inhalation, skin contact, or oral intake. Low-level TMA exposure can result in ocular, nasal, and respiratory irritation. Extended contact may lead to cytotoxicity and weakened immune responses, which clearly pose a risk to human health [4]. However, during the early stage of spoilage, the TMA concentration is still at a low level and is susceptible to interference from other volatile organic compounds in the aquatic product matrix, as well as environmental humidity and temperature, making accurate detection with high specificity and sensitivity a serious challenge [5]. Therefore, it is of great practical importance and urgent application need to develop a detection method. This method should be capable of sensitively identifying low-concentration TMA during the early stage of spoilage. It must also possess excellent anti-interference ability. Such a technique would enable early warning and accurate assessment of aquatic product freshness, thereby helping to ensure food safety.

Currently, detection methods for TMA mainly include gas chromatography [6], spectrophotometry [7], chemiluminescence [8], and gas sensor technology [9,10]. Among these, although gas chromatography and chemiluminescence have high sensitivities, they are limited by expensive equipment and complex operation, preventing their use in rapid field testing. Spectrophotometry is susceptible to interference from other amine gases and suffers from a long response time. However, micro-electromechanical system (MEMS) gas sensors present several benefits, including low cost, rapid response, and ease of integration [11]. In addition, MEMS heaters have low thermal mass and consume low power (e.g., <50 mW in pulsed mode), enabling portable use [12]. With proper insulation, they are safe for food applications. These features make them highly promising for the rapid evaluation of the freshness of aquatic products. The working principle of this sensor type relies on the interaction between the target gas and the sensing material. As gas molecules reach the material surface, processes of adsorption and desorption take place. These processes lead to a change in electrical resistance. By measuring this resistance variation, the concentration of the target gas can be determined quantitatively [13,14]. Although various materials are used for TMA detection, existing studies still have several limitations. For example, Liu et al. [15] synthesized α-Fe_2_O_3_ nanoparticles, but the response/recovery time was as long as 60 s/24 s, indicating slow response and recovery, and the detection limit was only 1 ppm. Wang et al. [16] prepared spherical V_2_O_5_ hierarchical structures, which exhibited a response value of only 2.8 at 100 ppm TMA, showing low sensitivity, and the detection limit was as high as 10 ppm, making it difficult to meet the requirements for low-concentration detection. Cheng et al. [5] constructed a Ce-MOF-808-GO-based QCM sensor, whose response decreased significantly when the relative humidity exceeded 35%, as water molecules tended to compete for adsorption active sites, causing signal interference and poor humidity resistance, which made it difficult to operate stably in high-humidity real-world food storage and transportation environments. In contrast, the morphology-controlled SnO_2_ hollow nanospheres synthesized in this work exhibit high sensitivity, a low detection limit, and good selectivity, and are capable of effectively detecting trimethylamine in high-humidity environments.

For MEMS gas sensors, the sensing material is the key factor that determines their performance [17]. Among various gas-sensing materials, SnO_2_ is a typical n-type semiconducting metal oxide. It offers good chemical stability, high electron mobility, and a sensitive response to reducing gases. As a result, it has been widely used in gas sensing applications [18,19,20]. However, conventional SnO_2_ materials have obvious limitations in sensing performance. They struggle to meet practical demands for high sensitivity, high selectivity, and fast detection. It is well recognized that the microstructure, crystal structure, and surface properties of sensing materials play key roles in determining their performances [21]. Morphology engineering not only increases the specific surface area, providing more active sites for gas adsorption, but also refines surface adsorption energy and charge transport pathways. These factors together contribute to better gas-sensing performance [22]. For example, Ge et al. [23] found that ZnFe_2_O_4_ hollow microspheres made of nanoparticles showed a higher response to isopropanol than those made of nanosheets. This was mainly due to their larger specific surface area and wider pore size, which allowed faster gas diffusion and more active reaction sites. Zhu et al. [24] synthesized α-Fe_2_O_3_ hollow nanocube sensors. These devices had a response/recovery time of only 9 s/11 s, outperforming the 13 s/24 s observed for solid cubes. Their detection limit reached as low as 1 ppm. The improved performance was attributed to the hollow structure, which effectively promoted the diffusion and release of TMA molecules, thus enhancing molecular transport efficiency. Therefore, carefully designing the morphology and structure of sensing materials provides an effective strategy for achieving superior gas-sensing performance.

In this work, a simple hydrothermal method was used to successfully synthesize SnO_2_ hollow nanospheres and SnO_2_ solid nanospheres. Based on these materials, MEMS gas sensors were constructed for the rapid detection of TMA in aquatic products, achieving both high sensitivity and good selectivity. Adsorption performance evaluation showed that the SnO_2_ hollow nanosphere sensor exhibited the highest TMA adsorption capacity (0.81 wt%) and the fastest adsorption and desorption rates (0.081/0.041 wt%·s^−1^). Gas sensing tests revealed the optimal performance of all materials at the 300 °C operating temperature. Among these materials, the SnO_2_ hollow nanospheres exhibited the highest response to TMA (10.5@100 ppm), along with good selectivity, reproducibility, and long-term stability. Furthermore, systematic analysis of the microstructure and surface chemistry of these sensing materials was conducted using multiple characterization techniques. Based on these results, the underlying gas sensing mechanism was thoroughly investigated. This study complements existing research perspective for enhancing the gas-sensing performance of sensing materials and provides a novel technical route for the rapid and effective assessment of aquatic product freshness.

## 2. Materials and Methods

### 2.1. Materials and Reagents

All analytically pure chemicals and solvents were directly used without additional purification, exactly as received. K_2_SnO_3_·3H_2_O (95%), urea (99%), SnCl_4_·5H_2_O (98%), NaOH (96%), absolute ethanol (99.9%), and hydrochloric acid (36%) were purchased from Sinopharm Chemical Reagent Co., Ltd. (Shanghai, China).

### 2.2. Synthesis of SnO_2_ Hollow Nanospheres

A hydrothermal method was adopted to prepare SnO_2_ hollow nanospheres [25]. In a typical synthesis, 0.384 g of K_2_SnO_3_·3H_2_O and 0.48 g of urea were thoroughly combined. Subsequently, this mixture was gradually introduced into a solvent system consisting of 50 mL of distilled water and 30 mL of absolute ethanol. The solution was stirred continuously until the solid materials became completely dissolved, forming a well-dispersed precursor solution. This resulting liquid was then transferred into a 100 mL Teflon-lined stainless steel autoclave. After being sealed, the autoclave was placed in an oven at 200 °C and maintained there for 24 h. Upon completion of the reaction, the autoclave was left to cool down naturally to ambient temperature. The white precipitate that formed was collected by centrifugation and rinsed repeatedly with absolute ethanol so as to remove impurities and any unreacted precursors. After the rinsing steps, the resulting product was allowed to dry overnight in an oven with a constant temperature set at 60 °C.

### 2.3. Synthesis of SnO_2_ Solid Nanospheres

A hydrothermal method was employed to prepare SnO_2_ solid nanospheres [26]. In a typical procedure, 0.351 g of SnCl_4_·5H_2_O and 4.00 g of NaOH were dissolved into 50 mL of a 50% (*v*/*v*) aqueous ethanol solution. This particular solvent system was created by combining 25 mL of absolute ethanol with 25 mL of deionized water. Following magnetic stirring for 5 min, the solid materials became completely dissolved, resulting in a transparent and colorless precursor solution. This solution was subsequently transferred into a 100 mL Teflon-lined autoclave. The autoclave was then sealed and positioned inside an oven at 200 °C for a period of 24 h. After the autoclave had cooled down naturally to room temperature, the precipitate that formed was collected by means of centrifugation. It was subsequently rinsed alternately with deionized water and absolute ethanol to eliminate any residual ionic impurities. In the final step, the resultant product was dried at 60 °C for 12 h, yielding solid SnO_2_ nanosphere powder.

### 2.4. Instruments and Characterization

To characterize the as-synthesized materials, a series of analytical techniques were applied. X-ray diffraction (XRD, Rigaku Ultimate IV, Rigaku Corporation, Tokyo, Japan) with Cu Kα radiation (λ = 1.5418 Å) at 25 °C was used to determine the phase composition and crystalline structure of the SnO_2_ nanomaterials. Scanning electron microscopy (SEM, Gemini SEM 500, Carl Zeiss AG, Oberkochen, Germany) and transmission electron microscopy (TEM, FEI Tecnai G20, FEI Company, Hillsboro, OR, USA) were employed to examine the morphological features, including the size, shape, and hollow/solid structures of the samples. Surface area measurements based on the BET method were conducted on a BELSORP-mini II analyzer (BEL Japan, Inc., Osaka, Japan) at 77 K with N_2_ as the probe gas to determine the specific surface area and pore size distribution, which are critical for gas adsorption. Diffuse reflectance spectra in the solid-state UV–vis range were obtained using a Shimadzu UV-VIS 2550 spectrophotometer (Kyoto, Japan) equipped with an integrating sphere over the range of 200–800 nm to evaluate the optical bandgap of the SnO_2_ nanomaterials. X-ray photoelectron spectroscopy (XPS, PHI-5000CESCA, ULVAC-PHI, Inc., Chigasaki, Kanagawa, Japan) with an Al Kα X-ray source (1486.6 eV) was used to analyze the surface chemical composition, elemental valence states, and oxygen vacancy-related defects of the materials.

### 2.5. Sensor Fabrication

This study employed a MEMS sensor integrating a microheater and interdigitated electrodes (Figure S1) [27,28]. The microheater provides thermal activation to the sensing material, while the interdigitated electrodes detect resistance changes caused by gas adsorption and desorption [29]. The gas-sensing material was thoroughly ground with absolute ethanol in an agate mortar, producing a homogeneous paste-like suspension. A fine brush was then used to evenly coat this slurry onto the electrode surface of the MEMS chip. After coating, the sensor was dried and subsequently thermally aged at 300 °C. This step improved interfacial adhesion stability and ensured reliable device operation. Real-time resistance changes in the sensor were monitored using a static testing system, allowing evaluation of its gas-sensing properties. The operating temperature could be adjusted by controlling the heating voltage. All gas-sensing tests were conducted at 45% RH (relative humidity) and at 25 °C. Humidity was regulated with a humidifier, and temperature was controlled by an air conditioning system. To ensure reproducibility, each experimental condition was tested at least three times.

### 2.6. Determination of TVB-N Content

TVB-N content was determined according to GB 5009.228-2016 [30] using an automatic Kjeldahl nitrogen analyzer [31]. Briefly, 10 g of sea bass muscle was mixed with 100 mL of distilled water, shaken for 30 min, and filtered. Then, 5 mL of the filtrate was distilled with 1.0 g of MgO solid. The distillate was automatically absorbed in 30 mL of 2% boric acid solution and titrated with 0.1000 mol/L HCl. Each sample was measured in triplicate.

## 3. Results

### 3.1. Characterizations of Materials

**Figure 1:**
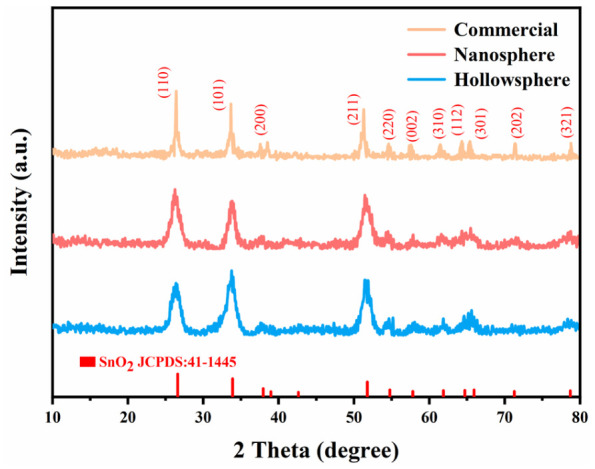
XRD patterns of commercial SnO_2_ nanoparticles, SnO_2_ solid nanospheres and SnO_2_ hollow nanospheres.

XRD analysis was performed to determine the crystal structures of the three samples, namely SnO_2_ hollow nanospheres, SnO_2_ solid nanospheres, and commercial SnO_2_ nanoparticles. Representative diffraction patterns are presented in Figure 1. All three materials exhibited sharp characteristic diffraction peaks, indicating their excellent crystallinity. The diffraction peak positions of all samples were in full agreement with the standard card for rutile SnO_2_ (JCPDS No. 41-1445) [32], and no impurity diffraction peaks were detected, confirming that all products were phase-pure SnO_2_.

**Figure 2:**
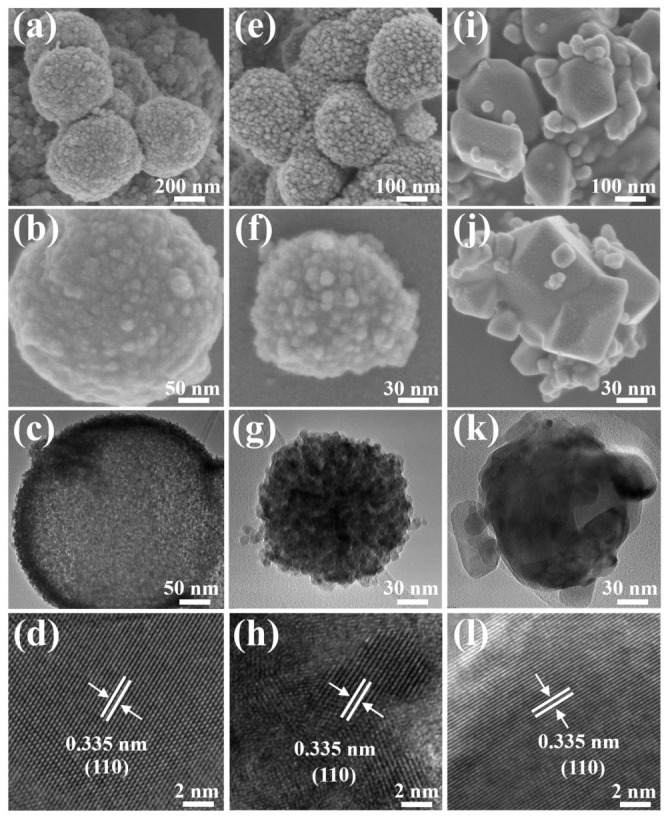
SEM, TEM and HRTEM images of different SnO_2_ nanomaterials: (**a**–**d**) SnO_2_ hollow nanospheres, (**e**–**h**) SnO_2_ solid nanospheres, and (**i**–**l**) commercial SnO_2_ nanoparticles.

Further analysis of the material morphology and structure was carried out via SEM and TEM. As illustrated in Figure 2a–c, the SnO_2_ hollow nanospheres exhibited a homogeneous morphology, with diameters falling between 300 and 400 nm. Stacked nanoparticles formed the surface, and the interior revealed a hollow cavity. Figure 2e–g display SnO_2_ solid nanospheres with diameters of 150–200 nm, which were well-dispersed and consist of solid spheres formed by particle accumulation. In contrast, commercial SnO_2_ nanoparticles showed an irregular, disordered polyhedral structure (Figure 2i–k). Figure 2d,h,l presented the high-resolution TEM images of the three materials. A lattice fringe spacing of approximately 0.355 nm was observed for all materials, corresponding to the (110) plane of tetragonal rutile SnO_2_. These results confirm the excellent crystallinity of the synthesized materials, corroborating the XRD results [33].

**Figure 3:**
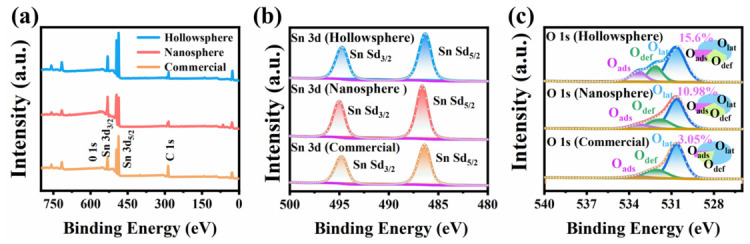
(**a**) XPS survey spectra of SnO_2_ hollow nanospheres, SnO_2_ solid nanospheres and commercial SnO_2_ nanoparticles. High resolution (**b**) Sn 3d and (**c**) O 1s XPS spectra of the three samples.

XPS analysis was conducted to examine the chemical states of the three samples. The survey spectrum (Figure 3a) revealed distinct signals corresponding to Sn 3d, O 1s, and C 1s, suggesting that the materials possess high purity [34]. For all three samples, the Sn 3d spectra (Figure 3b) exhibited the typical spin–orbit split doublet characteristic of Sn^4+^, which corresponds to the Sn 3d_3/2_ and Sn 3d_5/2_ orbitals, respectively [35]. The O 1s XPS spectra (Figure 3c) showed three well-resolved deconvoluted peaks, attributed to lattice oxygen (O_lat_), defect oxygen (O_def_), and adsorbed oxygen (O_ads_). Among these, lattice oxygen is structurally stable and not easily involved in gas-sensing reactions. In contrast, the chemisorbed oxygen species on the semiconductor surface show high chemical activity and can react with the target gas, modulating surface carrier density via redox reactions. Consequently, a higher fraction of adsorbed oxygen contributes favorably to the gas-sensing performance of the material [36,37]. Based on the deconvolution results, the adsorbed oxygen percentages for the three samples were determined and were presented as a pie chart in Figure 3c. The SnO_2_ hollow nanospheres had the highest proportion of adsorbed oxygen (15.6%), followed by SnO_2_ solid nanospheres (10.98%), and commercial SnO_2_ nanoparticles (3.05%). This discrepancy is primarily attributable to morphological variations, which affected specific surface area [22].

**Figure 4:**
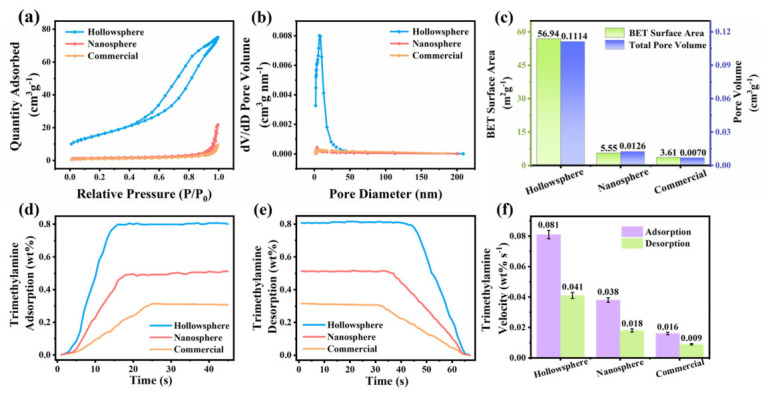
(**a**) Nitrogen adsorption–desorption isotherms, (**b**) pore size distributions, (**c**) BET surface area and pore volumes of SnO_2_ hollow nanospheres, SnO_2_ solid nanospheres, and commercial SnO_2_ nanoparticles. (**d**–**f**) Trimethylamine adsorption–desorption performance test of SnO_2_ hollow nanospheres, SnO_2_ solid nanospheres, and commercial SnO_2_ nanoparticles.

BET analysis was performed on the three samples to evaluate how morphology engineering influences their specific surface area and pore size. As shown in Figure 4a, the SnO_2_ hollow nanospheres exhibit a typical type IV isotherm with a distinct hysteresis loop, indicating a well-defined mesoporous structure [38]. In contrast, the SnO_2_ solid nanospheres and commercial SnO_2_ nanoparticles show a type III isotherm without an obvious hysteresis loop, indicating a non-porous or macroporous structure with low specific surface area [39]. Pore size distribution analysis (Figure 4b) revealed that the pores of SnO_2_ hollow nanospheres were mainly concentrated in the mesoporous range of 5–20 nm, showing a sharp peak. This indicated a narrow pore size distribution and excellent uniformity. In contrast, no distinct characteristic peaks were observed for SnO_2_ solid nanospheres or commercial SnO_2_ nanoparticles, further confirming their non-porous structure. As shown in Figure 4c, the SnO_2_ hollow nanospheres possessed the largest specific surface area (56.94 m^2^ g^−1^) and pore volume (0.1114 cm^3^ g^−1^), which were significantly larger than those of SnO_2_ solid nanospheres and commercial SnO_2_ nanoparticles. These results demonstrated that the construction of a hollow nanostructure significantly optimized the pore structure of SnO_2_, greatly increased its specific surface area and pore volume, and supplied abundant active adsorption sites and diffusion channels for gas molecules. This laid a structural foundation for its excellent gas-sensing performance [40]. The influence of morphology on gas adsorption–desorption performance was further investigated using a quartz crystal microbalance (QCM). The TMA adsorption–desorption behaviors of the three samples were presented in Figure 4d–f and Figure S2, which also provided the corresponding adsorption capacities and desorption rates for each material. The results showed that SnO_2_ hollow nanospheres exhibited the highest adsorption capacity for TMA (0.81 wt%), followed by SnO_2_ solid nanospheres (0.49 wt%), while the SnO_2_ hollow nanospheres had the fastest average adsorption and desorption rates (0.081 wt%·s^−1^ and 0.041 wt%·s^−1^, respectively), whereas commercial SnO_2_ nanoparticles exhibited the slowest rates. These findings align well with the data obtained from BET analysis: When the specific surface area is larger, a greater number of active sites become available for adsorption, which in turn leads to an enhancement in adsorption capacity. Meanwhile, the abundant mesoporous and hollow structures of SnO_2_ hollow nanospheres facilitate rapid gas diffusion, allowing TMA molecules to reach the adsorption sites more quickly. A larger specific surface area contributes to a greater number of active adsorption sites and thus a higher capacity. Meanwhile, the abundant mesoporous structure and hollow interior of the SnO_2_ hollow nanospheres accelerate gas diffusion, allowing TMA molecules to reach the active sites more rapidly [21].

### 3.2. Gas Sensitivity Performance

**Figure 5:**
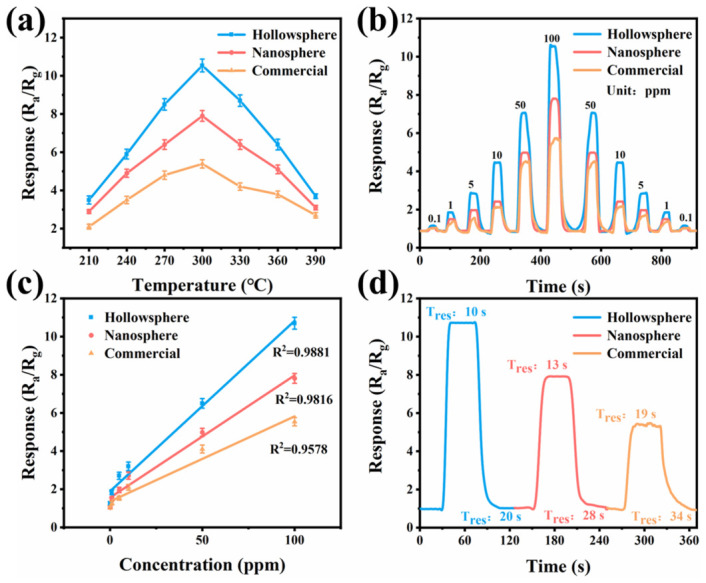
Sensing performance of the gas sensors based on SnO_2_ hollow nanospheres, SnO_2_ solid nanospheres and commercial SnO_2_ nanoparticles for trimethylamine. (**a**) The response of the gas sensor to 100 ppm trimethylamine at different operating temperatures (210–390 °C). (**b**) The dynamic response curves of the sensor to different concentrations of trimethylamine (0.1–100 ppm) at 300 °C. (**c**) The relationship between the response of the gas sensor and trimethylamine concentration at 300 °C. (**d**) The sensor’s response and recovery time curves to 100 ppm trimethylamine at 300 °C.

MEMS gas sensors were fabricated from the three SnO_2_ nanomaterials with distinct morphologies, and the TMA sensing properties of these SnO_2_ nanostructures were systematically evaluated. Measurements of the three sensors’ responses to 100 ppm TMA were carried out at operating temperatures varying from 210 °C to 390 °C. As shown in Figure 5a, all sensors reached their maximum response at 300 °C. The response gradually increased as the operating temperature rose to 300 °C, and then sharply decreased upon further temperature increase. Moreover, the SnO_2_ hollow nanospheres exhibited the highest sensitivity (R_a_/R_g_ = 10.5), followed by SnO_2_ solid nanospheres (R_a_/R_g_ = 7.8), while commercial SnO_2_ nanoparticles showed the lowest response (R_a_/R_g_ = 5.1). Compared with the solid architecture, the hollow structure possesses a larger specific surface area, which is more conducive to gas redox reactions and thus yields a higher sensitivity [41]. At 300 °C, the three sensors were then tested for their dynamic responses to TMA concentrations ranging from 0.1 to 100 ppm. Figure 5b clearly showed that as the TMA concentration increased from 0.1 ppm to 100 ppm, the response values of all three sensors increased accordingly; when the concentration decreased, the response values also decreased, indicating that these gas sensors possessed good reversibility and repeatability [22]. It was worth noting that a discernible response was observed for the SnO_2_ hollow nanosphere sensor even at a TMA level of only 0.1 ppm (Figure S3). For practical use, the quantitative dependence of the sensor signal on gas concentration, along with the response and recovery behavior, represented essential performance parameters [42]. Figure 5c demonstrated a favorable linear relationship between the response and TMA concentration, supporting the practical utility of the sensor for quantitative TMA detection (fitting equations are provided in Table S1) [43]. The response and recovery behavior of the three sensors toward 100 ppm TMA was illustrated in Figure 5d. Compared with SnO_2_ solid nanospheres (13 s/28 s) and commercial SnO_2_ nanoparticles (19 s/34 s), the SnO_2_ hollow nanospheres exhibited a faster response/recovery speed (10 s/20 s). The enhancement in sensing performance was ascribed to the hollow nanospheres’ larger specific surface area than the solid nanospheres, increasing the reactive area for the sensor. Meanwhile, their mesoporous structure also enabled target molecules to diffuse freely inside the SnO_2_ hollow nanospheres [44]. Consequently, under the same test conditions, the SnO_2_ hollow nanospheres gave both the highest response and the fastest response-recovery time for the target gas.

**Figure 6:**
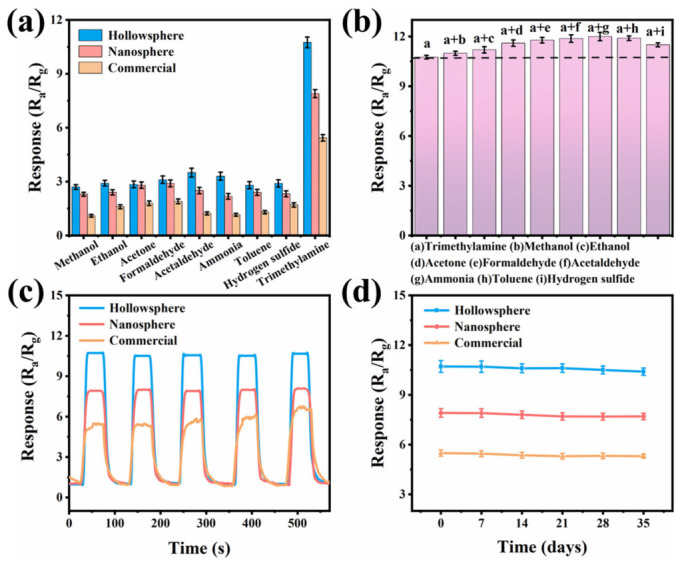
Gas-sensing properties of SnO_2_ hollow nanospheres, SnO_2_ solid nanospheres and commercial SnO_2_ nanoparticles. (**a**) Selectivity for 100 ppm trimethylamine and other interfering gases. (**b**) Response to mixed gases containing 100 ppm of trimethylamine and 100 ppm of each interfering gas. (**c**) Reproducibility and (**d**) long-term stability for sensing 100 ppm trimethylamine. All tests were conducted at 300 °C.

Selectivity is crucial for gas sensor applications [45,46]. At 300 °C, the three SnO_2_-based sensors were tested towards several common interfering gases of 100 ppm, including acetone, ethanol, methanol, formaldehyde, and ammonia, as shown in Figure 6a. Each sensor exhibited substantially stronger response to TMA than the interferents, with SnO_2_-based hollow nanospheres showing superior selectivity. Further investigation of anti-interference performance (Figure 6b) revealed that even when TMA coexisted with multiple interfering gases, the SnO_2_ hollow nanosphere sensor maintained excellent stability with a response fluctuation of less than 10%, demonstrating strong resistance to interfering gases and broad application prospects [47]. The sensor presented in this work is designed for rapid detection of TMA as the key spoilage marker, offering advantages of simple structure, fast response, and low cost. Future work may benefit from machine-learning-assisted sensing approaches [48,49] to discriminate target gas signals in complex food matrices. The three sensors were also subjected to repeatability and long-term stability assessments, as presented in Figure 6c,d. Five consecutive measurements of 100 ppm TMA under the same conditions confirmed that the sensors have good repeatability [50]. During one month of continuous testing, all sensors showed only slight fluctuations in their responses to 100 ppm TMA at 300 °C, indicating that the as-fabricated SnO_2_ nanomaterial-based sensors possess good long-term stability. As a key indicator of environmental adaptability, the humidity resistance was systematically evaluated through a gradient humidity experiment from 40% to 90%, and the influence of water molecules on sensor performance was analyzed. Figure S4 showed that despite the competitive adsorption between water molecules and the target gas on the sensor surface under elevated humidity, which led to a modest decrease in response, the maximum deviation was still confined to ±5% [51]. This excellent humidity tolerance was primarily attributed to the large specific surface area and abundant mesoporous structure of the hollow SnO_2_, which facilitated rapid adsorption and desorption of water molecules [37]. This inhibited excessive accumulation of surface hydroxyl groups and effectively mitigated the influence of humidity on baseline resistance and gas response. Furthermore, at the operating temperature of 300 °C, partial desorption of water molecules occurred, further reducing the interference of humidity on sensor performance [3]. This controllable humidity tolerance mechanism enables the sensor to operate stably in high-humidity environments, expanding its application range in humidity-sensitive fields such as the harvesting, processing, and transportation of aquatic products. Finally, Table S2 compares the TMA sensing performance of the SnO_2_ hollow nanosphere-based sensor developed in this study with that of previously reported MEMS sensors. As shown in the table, the SnO_2_ hollow nanosphere-based sensor exhibited higher sensitivity, faster response/recovery time, and a lower discernible concentration for TMA, demonstrating significant potential for practical applications.

### 3.3. Gas-Sensing Mechanism

**Figure 7:**
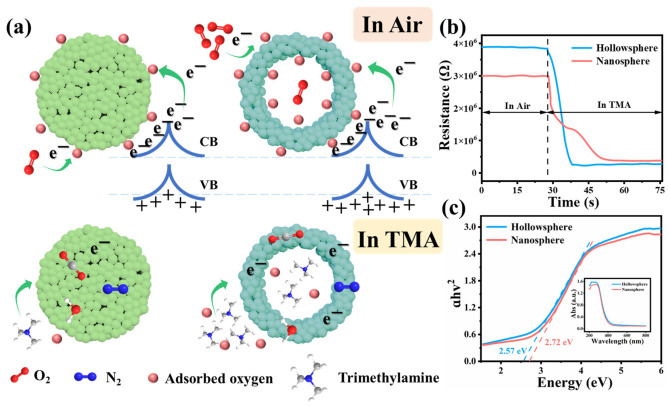
(**a**) Schematic illustration of the trimethylamine sensing mechanism of SnO_2_ hollow nanospheres and SnO_2_ solid nanospheres. (**b**) Resistance response curves of SnO_2_ hollow nanospheres and SnO_2_ solid nanospheres after trimethylamine exposure. (**c**) UV-vis diffuse reflectance spectra and Tauc curve of SnO_2_ hollow nanospheres and SnO_2_ solid nanosphere.

SnO_2_ is a classic n-type semiconductor, and its electrical conductivity is primarily governed by electrons occupying in the conduction band [18,52]. For SnO_2_-based gas sensors, the sensing mechanism follows a surface-controlled model [53], meaning that any resistance variation in the material is determined by the specific metal oxide and the surrounding gas composition. Figure 7a depicts how TMA is detected through surface-mediated reactions occurring between the sensing layer and incoming gas molecules. Upon exposure to ambient air, oxygen molecules extract electrons from the SnO_2_ conduction band, generating multiple types of chemisorbed oxygen ions [54]. At the same time, an electron-depleted layer forms at the surface of the sensing material reducing carrier concentration, thereby increasing the baseline resistance of the SnO_2_ nanostructure [55]. These processes are summarized by the following reactions:

(1) $${O_{2}}_{\left(gas\right)}\to {O_{2}}_{\left(ads\right)}$$

(2) $${O_{2}}_{\left(ads\right)}+e^{-}\to {O_{2}^{-}}_{\left(ads\right)}$$

(3) $${O_{2}^{-}}_{\left(ads\right)}+e^{-}\to {2O^{-}}_{\left(ads\right)}$$

(4) $${O^{-}}_{\left(ads\right)}+e^{-}\to {O^{2-}}_{\left(ads\right)}$$

When the SnO_2_ nanomaterial is brought into contact with TMA, a reducing gas, the chemisorbed oxygen species residing on its surface drive the oxidation of TMA molecules. As this oxidative process continues, the electrons, which have been previously trapped by the adsorbed oxygen, are released to the conduction band of the sensing material. This electron release alters the band structure, reduces the thickness of the electron depletion layer, and consequently lowers the material’s resistance. The overall surface reaction is described by the following equation [56]:

(5) $$2N{{\left({CH}_{3}\right)}_{3}}_{\left(ads\right)}\;+{\;21O}_{(ads)\;}^{-}\to \;9H_{2}O\;+\;6{\mathrm{CO}}_{2}\;+\;N_{2}\;+\;21e^{-}$$

Several factors account for the superior gas-sensing behavior of SnO_2_ hollow nanospheres, including their robust crystal structure and mesoporous hollow configuration. This hollow architecture, combined with a large specific surface area, supplies plentiful active sites across both the external surface and the interior cavity, facilitating oxygen adsorption and enabling the above reactions to proceed more efficiently. Under identical conditions, compared with solid nanospheres, the hollow counterparts return a greater number of electrons to the conduction band, which leads to a more pronounced change in resistance (Figure 7b) [21].

The optical bandgap was determined using the Tauc plot method according to the following equation [57]:

(6) $${\left(\alpha hv\right)}^{2}=A\left(hv-Eg\right)$$

where α is the absorption coefficient, h is Planck’s constant, v is the photon frequency, A is a constant, Eg is the optical bandgap, and the exponent n depends on the nature of the electronic transition (for SnO_2_, n = 1/2 for a direct allowed transition). The bandgap width of SnO_2_ solid nanospheres was measured to be 2.72 eV by UV–vis diffuse reflectance spectroscopy (Figure 7c), whereas that of SnO_2_ hollow nanospheres was only 2.57 eV. A reduced bandgap may facilitate electron transition from the valence band to the conduction band [57]. Consequently, a substantial quantity of charge carriers can be more easily injected into the conduction band of the SnO_2_ hollow nanospheres, resulting in a more pronounced variation in the material’s electrical conductivity [58]. Thus, the sensor based on SnO_2_ hollow nanospheres exhibits superior gas-sensing performance toward TMA.

### 3.4. Practical Application

**Figure 8:**
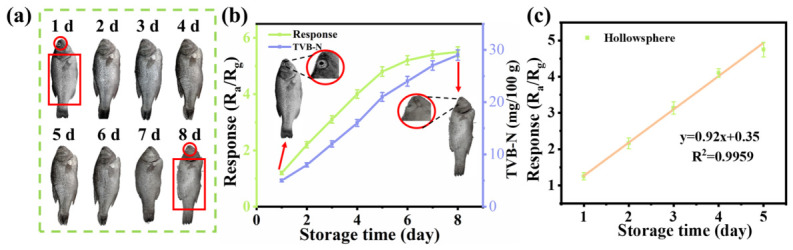
(**a**) Changes in sea bass stored at 0–4 °C for 1–8 days. (**b**) Changes in SnO_2_ hollow nanospheres sensor response (left axis) and TVB-N content (right axis) of sea bass during storage at 0–4 °C for 0–8 days. (**c**) Linear relationship between the response of the SnO_2_ hollow nanospheres sensor and storage time (first 5 days) at 0–4 °C.

With the aim of evaluating the practical performance of the SnO_2_ hollow nanosphere-based MEMS sensor, chilled sea bass (approximately 500 g) was selected as a sample to detect the TMA concentration released during refrigerated storage (0–4 °C) [25]. Figure 8a records the quality changes in sea bass stored at 0–4 °C for 1–8 days. During the first 1–4 days of storage, the surface gloss of the fish gradually diminished, the tail shape remained intact, with slightly turbid eyes. During 5–8 days of storage, the fish skin gradually became sticky, the scales loosened, the eye turbidity significantly intensified, and the abdomen became distended, with spoilage characteristics becoming markedly more severe as storage time extended. As shown in Figure 8b, both the sensor response and the total volatile basic nitrogen (TVB-N) content increased progressively with storage time. The TVB-N content, a widely recognized standard indicator for meat and aquatic product freshness [59,60], increased from an initial value of 5 mg/100 g to 29 mg/100 g after 8 days, which directly corresponded to the spoilage degree of sea bass and the rising sensor response. This correlation confirms the reliability of the sensor for freshness assessment. Further analysis revealed that under 0–4 °C conditions, the sensor response was linearly correlated with storage time (first 5 days), with R^2^ = 0.9959. Notably, when the storage time exceeded 5 days, the response value exceeded 4.5, corresponding to a TMA concentration of 10 ppm, indicating an early spoilage stage [61]. The gradual increase in response accurately reflects the continuous accumulation of TMA, a microbial metabolite, during the spoilage process. It should be noted that the current validation was performed only on sea bass, and further studies on other aquatic products are needed. Therefore, the MEMS sensor based on SnO_2_ hollow nanospheres offers broad application prospects for real-time and rapid assessment of aquatic product freshness.

## 4. Conclusions

In this study, characteristic SnO_2_ hollow nanospheres and SnO_2_ solid nanospheres are successfully synthesized via a hydrothermal reaction. Compared with commercial SnO_2_ nanoparticles, their gas-sensing behaviors influenced by morphological differences are systematically investigated. XRD, BET, TEM, and XPS are employed to characterize the prepared materials, revealing their crystalline phase, specific surface area, morphology, and elemental composition. According to gas-sensing analysis, the SnO_2_ hollow nanosphere sensor shows a high response (10.5@100 ppm), rapid response/recovery (10 s/20 s), favorable selectivity, and a discernible response at 0.1 ppm. The superior sensitivity of the SnO_2_ hollow structure is mainly attributed to its larger specific surface area providing abundant reaction sites for target molecules, significantly amplifying the resistance change in the sensor, while also facilitating efficient gas adsorption and desorption. Furthermore, the sensor is successfully applied to the reliable detection of TMA in sea bass, showing promising potential for practical application. This work presents a rapid and field-deployable method for assessing aquatic product freshness, with great significance for food safety.

## Data Availability

The original contributions presented in the study are included in the. article/Supplementary Materials, further inquiries can be directed to the corresponding authors.

## Supplementary Materials

The following supporting information can be downloaded at: https://www.mdpi.com/article/10.3390/foods15122123/s1, Figure S1: (a) Measuring circuit of MEMS gas sensor. (b) The panoramic views of the MEMS gas sensor test system. (c) The exploded views of the MEMS gas sensor.; Figure S2: Maximum adsorption capacity of SnO_2_ hollow nanospheres, SnO_2_ solid nanospheres, and commercial SnO_2_ nanoparticles for trimethylamine; Figure S3: Limit of detection of SnO_2_ hollow nanospheres toward 0.1 ppm trimethylamine at 300 °C; Figure S4: Response of SnO_2_ hollow nanospheres-based sensors to 100 ppm trimethylamine at different humidity levels; Table S1: Fitting equations for the relationship between the response of gas sensors and trimethylamine concentration; Table S2: Comparison of gas-sensing performance of MEMS sensors toward trimethylamine. References [62,63,64,65,66] are cited in the supplementary materials.

## Abbreviations

The following abbreviations are used in this manuscript:

BET	Brunauer–Emmett–Teller
HRTEM	High-resolution transmission electron microscopy
MEMS	Micro-Electro-Mechanical Systems
O_lat_	Lattice oxygen
O_ads_	Adsorbed oxygen
O_def_	Defect oxygen
QCM	Quartz crystal microbalance
SEM	Scanning electron microscopy
SnO_2_	Tin dioxide
TEM	Transmission electron microscopy
TMA	Trimethylamine
UV-Vis	Ultraviolet–visible
XPS	X-ray photoelectron spectroscopy
XRD	X-ray diffraction
