# Supplementary material for: Rapid Non-Destructive Assessment of Aquatic Products Freshness by Gas Sensor Based on Morphology-Controlled SnO2 Hollow Nanosphere
Source: Foods. 2026 Jun 12;15(12):2123. doi: 10.3390/foods15122123 (PMC13298068; doi:10.3390/foods15122123)
Supplement: Supplementary file 1 [file foods-15-02123-s001.zip › foods-4304152-supplementary.pdf]

Supplementary Material

Rapid Non-Destructive Assessment of Aquatic Products Freshness by Gas Sensor based on Morphology-Controlled SnO_2 Hollow Nanosphere

Han Liu[1,†]; Yingkun Dong[1,†]; Haixia Zhou[1]; Weihao Wu[1]; Ziliang Fan[1]; Cheng Zhao[1,2*]; Yongheng Zhu[1*]

1 College of Food Science and Technology, Shanghai Ocean University, Shanghai 201306, China; m230351145@st.shou.edu.cn (H.L.); 19818505418@163.com (Y.D.); d250400135@st.shou.edu.cn (H.Z.); d240400095@st.shou.edu.cn (W.W.); 18888820272@163.com (Z.F.);

2 Henan Railway Food Safety Management Engineering Technology Research Center, Zhengzhou Railway Vocational & Technical College, Zhengzhou 450000, China;

* Correspondence: allenz_1222@foxmail.com; zhuyh7862@126.com

† These authors contributed equally to this work.

Figure and Table caption:

Figure S1. (a) Measuring circuit of MEMS gas sensor. (b) The panoramic views of the MEMS gas sensor test system. (c) The exploded views of the MEMS gas sensor.

Figure S2. Maximum adsorption capacity of SnO_2 hollow nanospheres, SnO_2 solid nanospheres, and commercial SnO_2 nanoparticles for trimethylamine.

Figure S3. Limit of detection of SnO_2 hollow nanospheres toward 0.1 ppm trimethylamine at 300℃.

Figure S4. Response of SnO_2 hollow nanospheres-based sensors to 100 ppm trimethylamine at different humidity levels.

Table S1: Fitting equations for the relationship between the response of gas sensors and trimethylamine concentration.

Table S2: Comparison of gas-sensing performance of MEMS sensors toward trimethylamine.

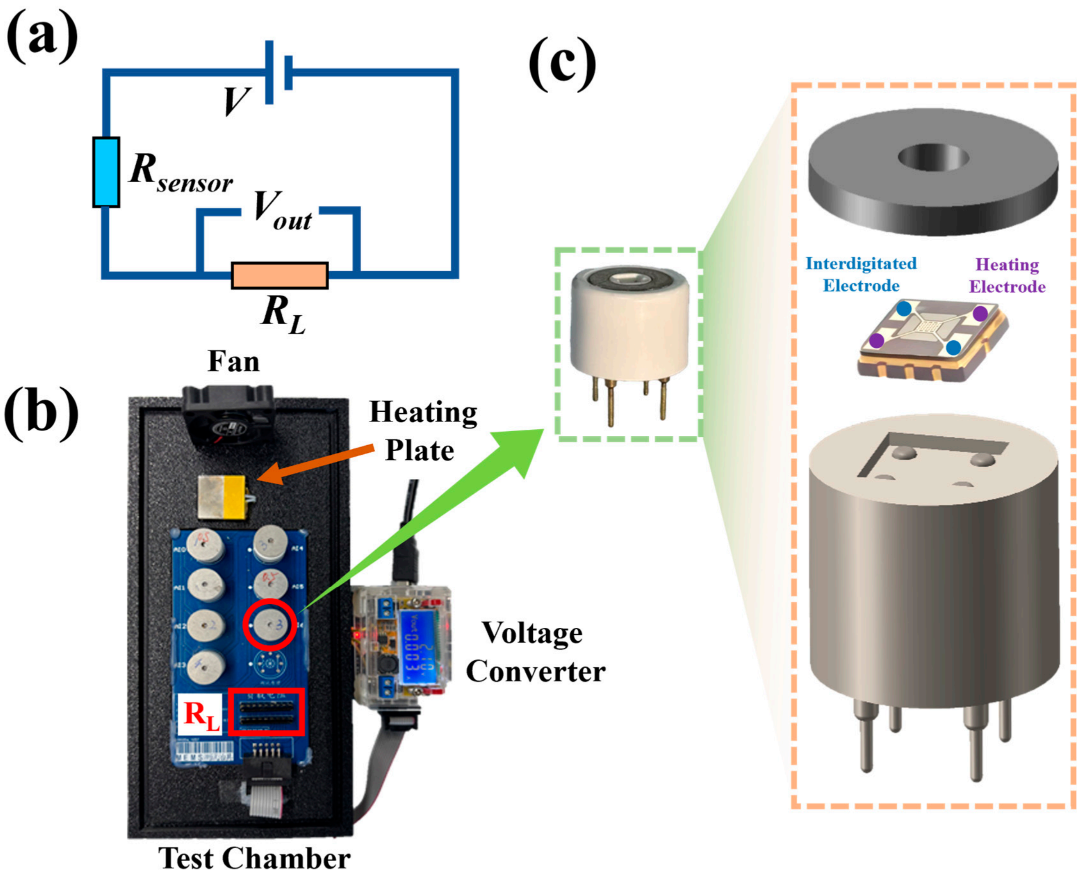

Figure S1. (a) Measuring circuit of MEMS gas sensor. (b) The panoramic views of the MEMS gas sensor test system. (c) The exploded views of the MEMS gas sensor.

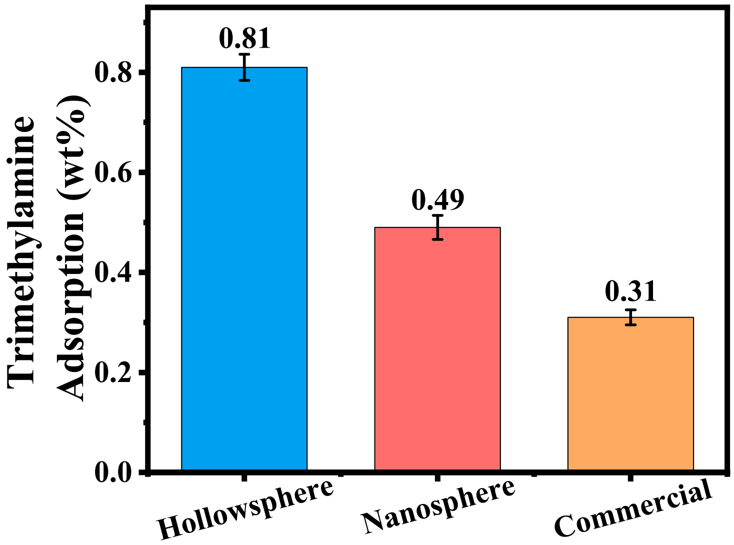

Figure S2. Maximum adsorption capacity of SnO_2 hollow nanospheres, SnO_2 solid nanospheres, and commercial SnO_2 nanoparticles for trimethylamine.

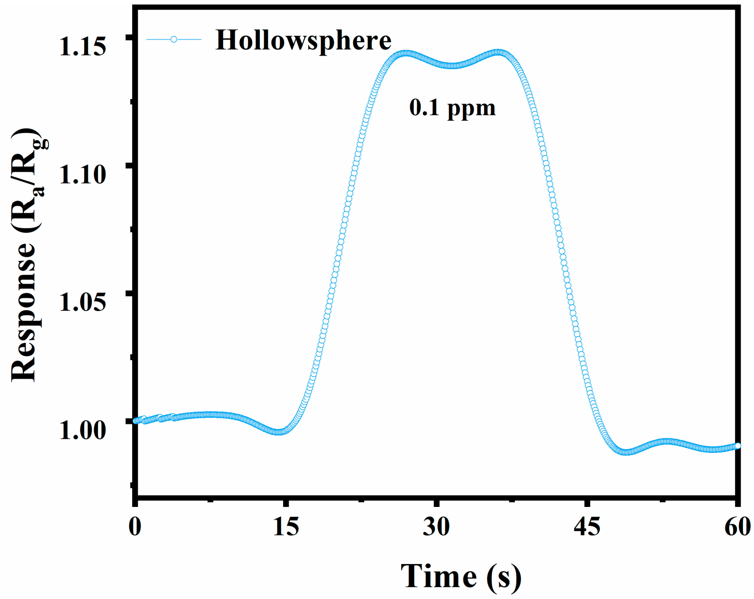

Figure S3. Limit of detection of SnO_2 hollow nanospheres toward 0.1 ppm trimethylamine at 300℃.

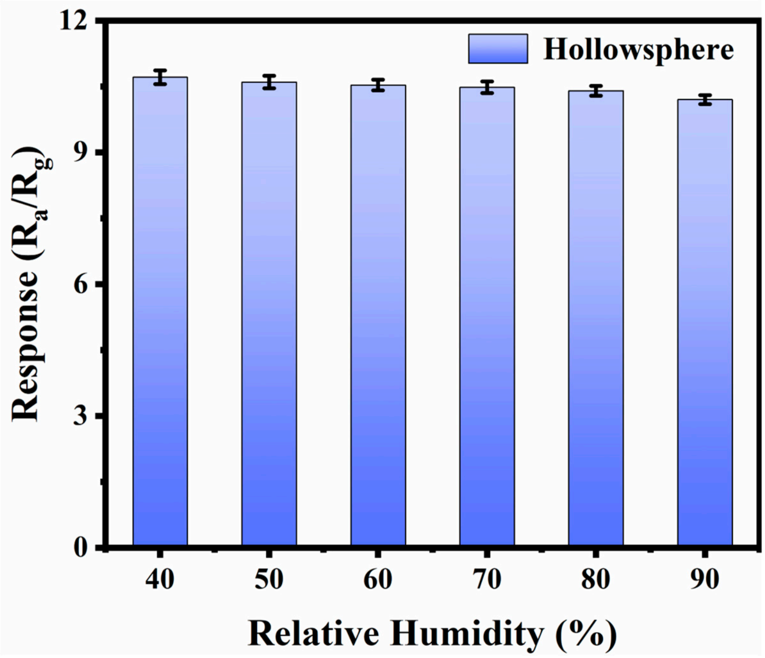

Figure S4. Response of SnO_2 hollow nanospheres-based sensors to 100 ppm trimethylamine at different humidity levels.

Table S1: Fitting equations for the relationship between the response of gas sensors and trimethylamine concentration

Materials	Fitting curve equation	R^2
SnO_2 hollow nanospheres	y=0.08933x+1.89358	0.9881
SnO_2 solid nanospheres	y=0.0641x+1.56057	0.9816
commercial SnO_2 nanoparticles	y=0.04459x+1.36048	0.9578

Table S2: Comparison of gas-sensing performance of MEMS sensors toward trimethylamine

Materials	Conc. (ppm)	Response (R_a/R_g)	Tres/Trec (s)	Detection limit (ppm)	Ref.
α-Fe_2O_3	20	6.3	9/11	1	[S1]
V_2O_5	100	2.8	5/28	10	[S2]
In_2O_3@In_2S_3	10	5.5	28/48	0.1	[S3]
Co_3O_4/In_2O_3	10	11.7	25/68	1	[S4]
E-g-C_3N_4/Bi_2MoO_6	20	10.6	42/209	1.3	[S5]
α-Fe_2O_3	100	27.8	60/240	1	[S6]
α-Fe_2O_3/ZnO	200	11.6	1/7	10	[S7]
Bi/$ZnFe_2O_4$	100	15.7	3/8	0.2	[S8]
SnO_2	100	10.5	10/20	0.1	This work

(S1) Zhu, K.; Zhu, Z.; Xu, S.; Zhao, C.; Ni, T. Controlled synthesis of α-Fe_2O_3 nanocubes for gas-sensing applications: Feasibility of assessing crucian carp (Carassius auratus) freshness via trimethylamine levels. *Food Chemistry* **2024**, *441*, 138361. DOI: 10.1016/j.foodchem.2024.138361.

(S2) Wang, D.; Gu, K.; Zhao, Q.; Zhai, C.; Yang, T.; Lu, Q.; Zhang, J.; Zhang, M. Synthesis and trimethylamine sensing properties of spherical V_2O_5 hierarchical structures. *New Journal of Chemistry* **2018**, *42*(17), 14188-14193. DOI: 10.1039/C8NJ02506A.

(S3) Meng, D.; Qiao, T.; Ji, Y.; Wang, R.; Zhang, Y.; San, X. Hydrangea-like In_2O_3@In_2S_3 n-n heterostructures for high-efficiency TMA measurement. *IEEE Transactions on Instrumentation and Measurement* **2023**, *72*, 1-9. DOI: 10.1109/TIM.2023.3237223.

(S4) Ji, Y.; Zhang, N.; Xu, J.; Jin, Q.; San, X.; Wang, X. Co_3O_4/In_2O_3 p-n heterostructures based gas sensor for efficient structure-driven trimethylamine detection. *Ceramics International* **2023**, *49*(11), 17354-17362. DOI: 10.1016/j.ceramint.2023.02.103.

(S5) Wu, K.; He, X.; Ly, A.; Lahem, D.; Debliquy, M.; Zhang, C. Highly sensitive and selective gas sensors based on 2D/3D Bi_2MoO_6 micro-nano composites for trimethylamine biomarker detection. *Applied Surface Science* **2023**, *629*, 157443. DOI: 10.1016/j.apsusc.2023.157443.

(S6) Liu, L.; Fu, S.; Lv, X.; Yue, L.; Fan, L.; Yu, H.; Gao, X.; Zhu, W.; Zhang, W.; Li, X.; Zhu, W. A gas sensor with Fe_2O_3 nanospheres based on trimethylamine detection for the rapid assessment of spoilage degree in fish. *Frontiers in Bioengineering and Biotechnology* **2020**, *8*, 567584. DOI: 10.3389/fbioe.2020.567584.

(S7) Zhang, R.; Wang, L.; Deng, J.; Zhou, T.; Lou, Z.; Zhang, T. Hierarchical structure with heterogeneous phase as high-performance sensing materials for trimethylamine gas detecting. *Sensors and Actuators B: Chemical* **2015**, *220*, 1224-1231. DOI: 10.1016/j.snb.2015.07.036.

(S8) Zhao, X.; Liu, W.; Yang, X.; Xu, W.; Yao, L.; Sun, Z.; Pan, G.; Huang, L.; Cheng, Y. Synergistic effects of Bi doping in $ZnFe_2O_4$ nanoparticles for enhanced trimethylamine sensing via oxygen vacancy accumulation and catalytic activation. *Journal of Environmental Chemical Engineering* **2026**, 121141. DOI: 10.1016/j.jece.2026.121141.
